# Abnormal Retinal Vessel Architecture in Albinism and Idiopathic Infantile Nystagmus

**DOI:** 10.1167/iovs.63.5.33

**Published:** 2022-05-26

**Authors:** Shafak Toufeeq, Irene Gottlob, Zhanhan Tu, Frank A. Proudlock, Anastasia Pilat

**Affiliations:** 1 Ulverscroft Eye Unit, Department of Neuroscience, Psychology and Behaviour, University Of Leicester, University Road, Leicester, LE1 7RH, United Kingdom; 2 Oxford Eye Hospital, Level LG1 John Radcliffe Hospital, Headley Way, Headington, Oxford OX3 9DU, United Kingdom

**Keywords:** retinal vessel development, albinism, idiopathic infantile nystagmus syndrome (INS), color fundus imaging, vessel segmentation

## Abstract

**Purpose:**

Infantile nystagmus syndrome (INS) causes altered visual development and can be associated with abnormal retinal structure, to which vascular development of the retina is closely related. Abnormal retinal vasculature has previously been noted in albinism but not idiopathic infantile nystagmus. We compared the number and diameter of retinal vessels in participants with albinism (PWA) and idiopathic infantile nystagmus (PWIIN) with controls.

**Methods:**

Fundus photography data from 24 PWA, 10 PWIIN, and 34 controls was analyzed using Automated Retinal Image Analyzer (ARIA) software on a field of analysis centered on the optic disc, the annulus of which extended between 4.2 mm and 8.4 mm in diameter.

**Results:**

Compared with controls, the mean number of arterial branches was reduced by 24% in PWA (15.5 vs. 20.3, *P <* 0.001), and venous branches were reduced in both PWA (29%; 12.9 vs. 18.2, *P <* 0.001) and PWIIN (17%; 15.1 vs. 18.2, *P =* 0.024). PWA demonstrated 7% thinner “primary” (before branching) arteries (mean diameter: 75.39 µm vs. 80.88 µm, *P =* 0.043), and 13% thicker (after branching) “secondary” veins (66.72 µm vs. 59.01 µm in controls, *P =* 0.009).

**Conclusions:**

PWA and PWIIN demonstrated reduced retinal vessel counts and arterial diameters compared with controls. These changes in the superficial retinal vascular network may be secondary to underdevelopment of the neuronal network, which guides vascular development and is also known to be disrupted in INS.

Infantile nystagmus syndrome (INS) is defined as rhythmic, involuntary oscillations of the eyes^1^ which develop within the first 6 months of life.^2^ It can be associated with chiasmal conditions, such as albinism, retinal disease, such as rod and cone dystrophies, and fusional maldevelopment nystagmus syndrome caused by infantile squint syndrome.^3^ Idiopathic INS (IIN) has historically been a diagnosis of exclusion, but more recently has shown to be heritable in an X-linked manner in association with *FRMD7* mutations,^4^ for whom optical coherence tomography (OCT) studies have demonstrated changes in the retina and optic nerve head.^5^

Albinism refers to a group of heritable conditions characterized by hypopigmentation of the skin, hair, and eyes, for whom numerous genetic mutations have been identified. These include oculocutaneous forms (OCA1-8), purely ocular forms (OA1), or as part of syndromic conditions, such as Hermansky Pudlak syndrome (HPS1-11).^6^ Visual pathway anomalies include high refractive error,^7^ iris transillumination, foveal hypoplasia (FH), retinal nerve fiber layer thinning, optic nerve head dysmorphism, chiasmal misrouting, cortical changes, and nystagmus.^5^^,^^8^^,^^9^

Pigmentary deficits are caused by mutations affecting key enzymes across the melanin synthesis pathway. The mechanisms underlying pigmentation are complex. A sophisticated control mechanism exists with tyrosinase activity being modulated using the internal melanosome pH, which is in turn is determined by melanosome membrane potential controlled through a combination of ion channels.^10^ Consequently, mutations affecting either tyrosinase directly, or any of its control mechanisms, have the potential to influence pigmentation. It has been suggested that L-DOPA, an intermediate in the melanin synthesis pathway, may be important signaling molecule in the control of retinal development. For example, conditions such as Foveal hypoplasia-optic nerve decussation defect-anterior segment dysgenesis (FHONDA) syndrome display the albinism visual phenotype but have normal pigmentation, suggesting that under-expression of melanin may not be the underlying cause of delayed retinal development.^11^ In OCA1, mutations in the *TYR* gene cause impairment of the tyrosinase function of converting tyrosine to L-DOPA.^6^ Other mutations are thought to influence the pH internal melanosome environment under the control of a voltage dependent proton pump. In the case of OCA2, the gene codes for a chloride channel controlling the membrane potential of the melanosome.^10^ The OA1 gene codes for an L-DOPA receptor and indirectly reduces retinal melanin synthesis by disruption of the downstream signaling pathway.^12^

Many of these visual deficits stem back to delayed neurogenesis in the retina, possibly related to reduced expression of L-DOPA during visual development.^13^^,^^14^ Investigators have attempted to bridge the gap between the melanin synthesis pathway and visual development. Jeffery et al. (1997) showed that the insertion of a functional tyrosinase gene in transgenic albino mice and rabbits restored normal ganglion cell density in the central retina, and rod photoreceptor count.^15^ Jeffery (1998) further suggested this may relate to the action of L-DOPA as a regulator of mitotic activity in the developing retina, whose absence may cause delayed retinal maturation.^16^ Lopez et al. (2008) expanded on this by demonstrating the influence of L-DOPA, via modulation of OA1 in the retinal pigment epithelium (RPE), on pigment epithelium derived factor (PEDF) production. PEDF plays an important role in neurosensory retinal development.^12^

There is strong evidence that the development of neural and vascular systems share common genetic and signaling pathways,^17^ and normal retinal angiogenesis is dependent on neural guidance mechanisms.^18^^,^^19^ No studies to date have investigated retinal vasculature in IIN, and only limited research interest exists for albinism. Conley et al. (2016) described abnormal features of the optic disc and peripapillary vasculature, including mild optic nerve hypoplasia and central vessels emerging and extending nasally from the optic disc in albinism compared with controls.^20^ This was supported by findings of Neveu et al. (2005), who reported that retinal vasculature in albinism tends to be more asymmetric with a reduced number of arterial vessel branches.^21^ However, these studies lacked quantitative methods for analyzing vessel architecture, and did not correlate vessel morphology with clinical features such as foveal morphology.

In this study, we used retinal vessel analysis software to assess and compare the number and diameter of retinal vessels, in an annulus around the optic disc, in participants with albinism (PWA) and idiopathic infantile nystagmus (PWIIN) with controls. The data were also correlated with severity of FH.

## Materials and Methods

### Participants

This prospective observational study included the data of 24 PWA (15 men and 9 women, mean age ± SD: 34 ± 14 years) and 10 PWIIN (7 men and 3 women, mean age ± SD: 32 ± 17 years). Participants were recruited from the neuro-ophthalmology clinic at Leicester Royal Infirmary, UK. Approval to conduct the study was gained from the local ethics committee and informed consent from all participants was obtained with adherence to the tenets of the Declaration of Helsinki.

Diagnosis of PWA was confirmed by the presence of phenotypic features fulfilling criteria presented by Kruijt et al. (2018),^22^ who specify the diagnostic threshold as three “major” criteria (including significant ocular hypopigmentation, misrouting on visual evoked potential recordings, and grade 2 or more FH^23^), or two major criteria and two minor criteria (the latter including nystagmus, skin, or mild ocular hypopigmentation, and grade 1 FH). Diagnostic criteria for PWIIN were the presence of early onset nystagmus with characteristic nystagmus waveforms, good visual acuity, mild or absent FH on OCT (see Supplementary Fig. S1 for an illustration), normal electrodiagnostic testing and no other ophthalmic or neurological pathology. Genetic testing to determine subtype of albinism was not available for all study participants and so we assume the cohort is a combination of OA and OCA, and refer to the cohort as PWA. The control group included 34 gender-, ethnicity-, age-, and refraction-matched healthy controls.

All participants with ocular comorbidities and high refractive errors (myopia or hypermetropia of 5 Diopters or more in spherical equivalent), systemic comorbidities associated with retinal vascular diseases, such as systemic hypertension, chronic renal disease, diabetes, blood hyperviscosity, and anemia were excluded from the study.

Supplementary Table S1 shows the baseline demographic characteristics of the study participants. The male to female ratio was 1.7:1, 2.3:1, and 1.8:1 in the PWA, PWIIN, and control groups, respectively. The mean age was 33.8, 31.8, and 33.1 years, respectively.

**Figure 1.**
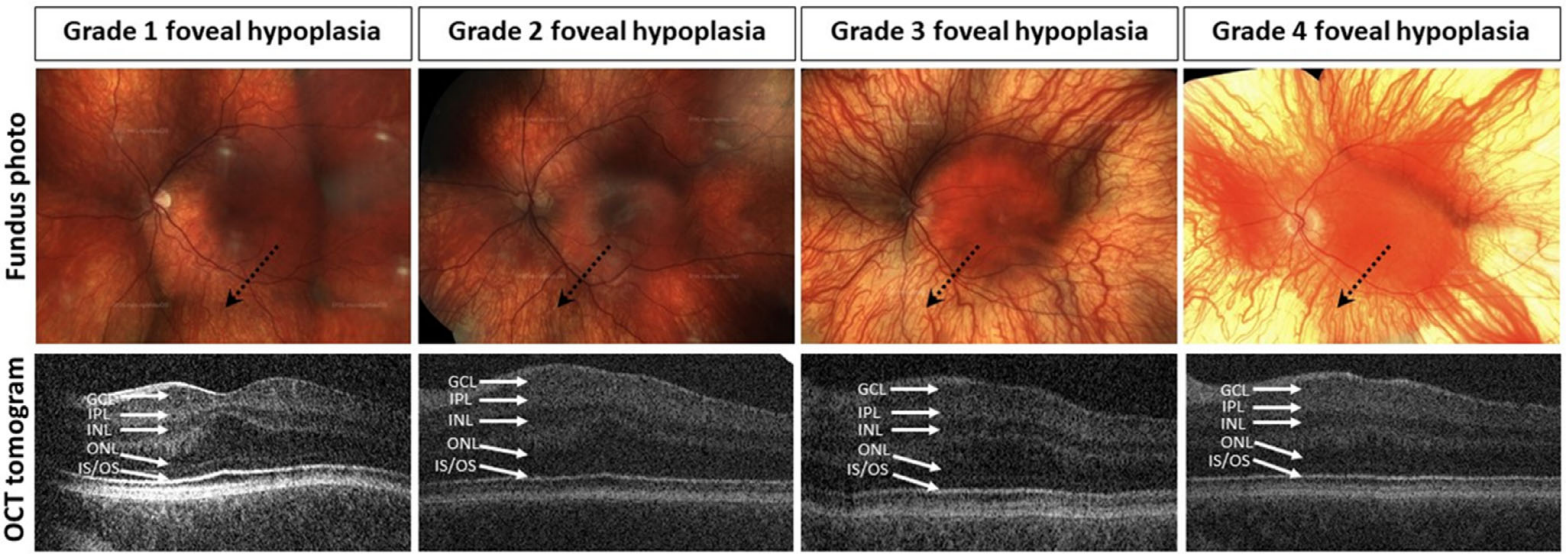
Full field color fundus images and horizontal macular OCT horizontal foveal B-scan images of PWA with differing grades of FH. Fundus photography (*top row*) shows: absent foveal reflex in all patients, partially (grades 1 and 2 FH) or fully (grades 3 and 4 FH) visible choroidal vessels (*dotted arrows*). OCT images (*bottom row*) show: small foveal pit, GCL/INL/IPL continuation with domed ONL, presence of IS/OS updrift in grade 1 FH; residual foveal pit, thicker continuous GCL/INL/IPL with minimal domed ONL and small IS/OS updrift in grade 2 FH; absent foveal pit, thick continuous GCL/INL/IPL/OPL, and thinned ONL in grades 3 and 4 FH, with minimal updrift of IS/OS in grade 3 and flat IS/OS in grade 4 patients with FH. OCT, ocular coherence tomography; GCL, ganglion cell layer; IPL, inner plexiform layer; INL, inner nuclear layer; OPL, outer plexiform layer; ONL, outer nuclear layer; IS/OS, = inner segment/outer segment junction; FH, foveal hypoplasia.

Complete ophthalmic examination was performed on all participants, including best-corrected visual acuity, refraction, orthoptic assessments, slit lamp examination, fundus and OCT imaging, and eye movement recordings for those with nystagmus. OCT imaging (Envisu 2300, Leica Microsystems, Germany; 840 nm wavelength. optical axial resolution in tissue <4 µm; 12 × 8 × 2 mm volumes comprised of 600 A scans and 80 B scans; 1.67 second acquisition time) was used by one investigator (author S.T.) to grade the degree of FH using the standardized foveal hypoplasia scale developed by Thomas et al. (2011).^23^ This has been illustrated in Figure 1.

Using 1% tropicamide eye drops for mydriasis, 45 degree color fundus pictures of both eyes were taken using a Zeiss VISUCAM^NM/FA^ (Oberkochen, Germany) fundus camera. A full field quadratic and feathered montage was created from the images using i2k RETINA software (standard version 2.4) from DualAlign. The eye with the higher quality fundus image was selected for the study.

### Retinal Vessel Detection and Analysis

Vessel detection and measurements were performed using the standardized settings of Automated Retinal Image Analyzer (ARIA), an open source, MATLAB (MathWorks, Inc., Natick, MA, USA) based program.^24^ Following automated vessel segment detection, this program additionally allows for visualization of retinal vessel wall detection, and manual removal of incorrectly identified vessel segments, such as that of choroidal vessels, and manual merging of remaining vessel segments into branches. Retinal vessel branches could be manually recognized and differentiated from choroidal vessels as retinal vessels follow an obvious visible route back to the optic nerve. This was performed by the same investigator (author S.T.), who was masked to the type of participant with random number allocated for each fundus photograph.

**Figure 2.**
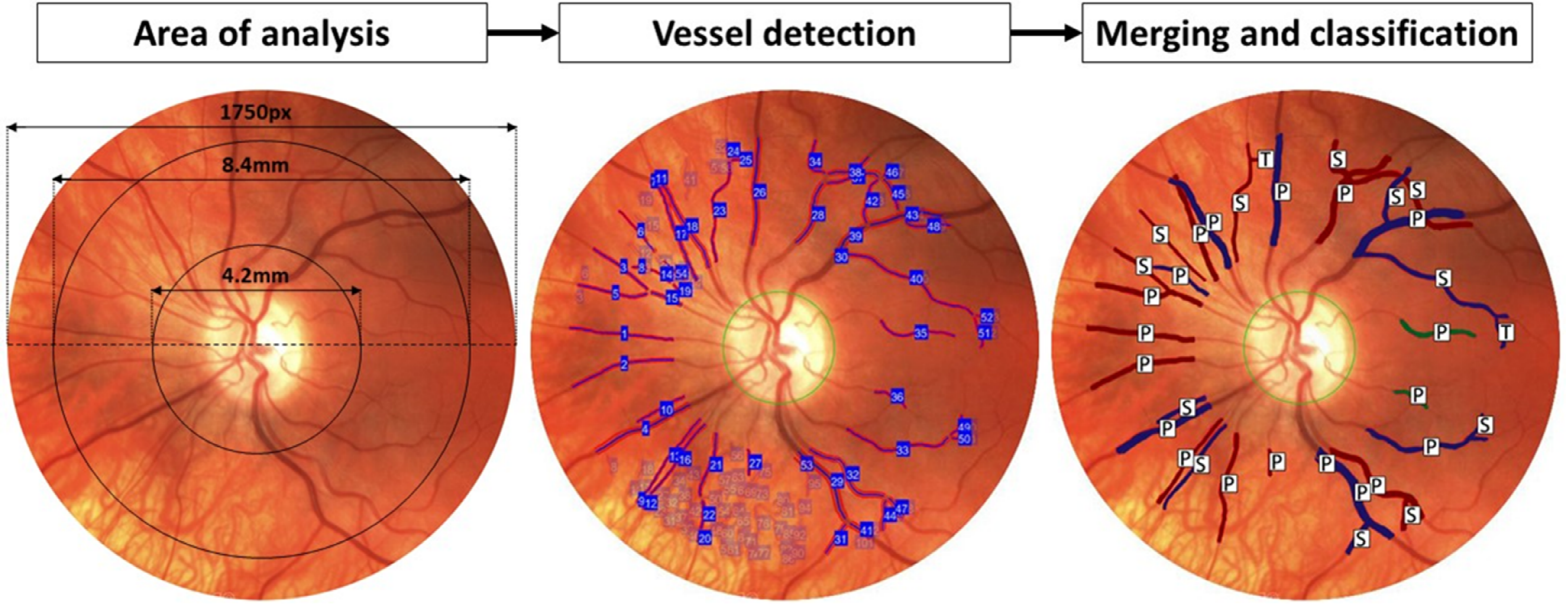
Recognition of retinal vessels in a study participant with albinism. Area of analysis: Initial image with illustration of 1750 pixel ring diameter centered on optic disc, and annular area of detection defined within rings 4.2 mm and 8.4 mm in diameter both centered on the optic disc. Automated vessel detection by ARIA program: Detection of vessel segments (numbers depicting choroidal vessels and other erroneously detected vessel segments which have been removed manually are shown faded for illustrative purposes). Merging and classification: Remaining vessel segments have been merged. arteries (marked *red*), veins (marked *blue*), and unclassified (marked *green*; excluded from the analysis) vessels have been labeled as primary (P), secondary (S), or tertiary (T).

The lack of pigmentation in some PWAs increased the prominence of choroidal vasculature, particularly beyond the major vascular arcades (see Supplementary Fig. S1). This greatly increased processing time and time taken to manually remove these detected segments. This was mitigated by using Adobe Photoshop software to crop the full field montage to a circular area of a 1750-pixel diameter centered on the optic disc. The field of analysis was then limited to an annulus centered on the optic disc, whose inner and outer margins extended 4.2 mm and 8.4 mm in diameter, respectively. Although choroidal vessels were still detected within this annulus, they were far fewer in number (see Fig. 2).

The vessels were manually classified by type (arterial or venous) and quadrant (superonasal, superotemporal, inferonasal, and inferotemporal, as well as the total). Vessels which could not be identified as arterial or venous were excluded (33% of participants averaging 1.9 excluded vessels each) from the analysis. For each of these categories, mean vessel quantity and diameter was then calculated separately for “primary” vessel, “secondary” vessel, and “tertiary” vessel branches, as well as their total, using a systematic ordering method; vessels emerging from the optic disc were classified as “primary,” and at branching points thereafter the thinner vessel branch was labeled a “secondary” vessel and the thicker branch would continue as the “primary” vessel. Tertiary vessels were derived from secondary vessels in the same manner (see Fig. 2).

As foveal changes are more frequently observed in PWA, the effect of degree of FH on vascular parameters was explored in this group. The 24 participants of the PWA group were divided into “moderate” FH (grade 1 *n* = 6 and grade 2 *n* = 6) and “severe” FH (grade 3 *n* = 2 and grade 4 *n* = 10) subgroups, and the analysis repeated in comparison to the control group.

### Retinal Vessel Width Measurement

Proprietary VISUPAC software from Zeiss was used to obtain a value for pixel width for each image. This software is pre-calibrated by the manufacturer to the telecentric optic to obtain the real size of the image, with adjustments for spherical equivalent and corneal radius^25^ (the latter was entered as 7.7 mm for all eyes as per the Schematic eye of Gullstrand).^26^ B-scans were scaled based on a pixel dimension of 2.4 µm × 20 µm in the axial and lateral planes, respectively. The pixel width was inserted into ARIA to obtain vessels diameter measurements in microns. The software was then used to calculate the mean, minimum, and maximum diameter of each vessel branch.

### Retinal Thickness Measurement

Where possible, retinal thickness was measured in the PWA group from the OCT B-scan images temporal to the optic nerve head, at a horizontal distance of 6.3 mm from the optic disc center. This represented mid-way between the inner and outer margins of the annulus used for the vessel analysis (from 4.2 mm to 8.4 mm)

### Statistical Analysis

SPSS software version 24.0 (SPSS, Inc., Chicago, IL, USA) was used. Vascular parameters were normally distributed. ANOVAs were used to compare mean vessel quantity and mean vessel diameter among the PWA, PWIIN, and control groups. Significant differences were explored using a post hoc analysis with *P* values adjusted using Bonferroni correction. *P* ≤ 0.05 was considered statistically significant.

Because albinism is associated with retinal nerve fiber layer thinning^9^ and a tendency toward higher refractive error,^7^ we correlated retinal thickness measurements and spherical equivalent to mean vessel count and mean vessel diameter measurements using Pearson's correlation.

## Results

### Vessel Count

#### Participants With Albinism

**Figure 3.**
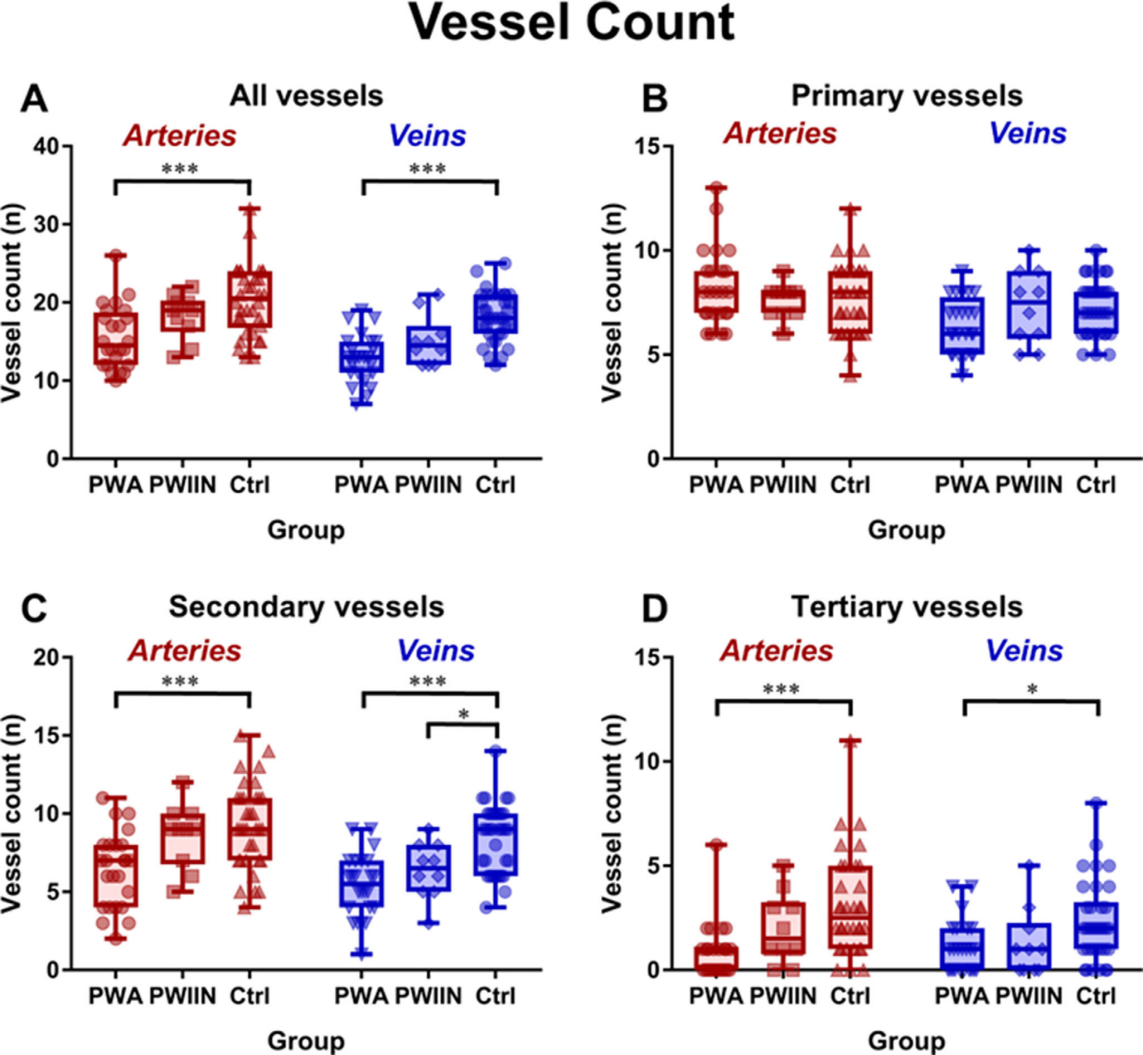
Box and whisker plots of participants with participants with albinism (PWA), participants with idiopathic infantile nystagmus (PWIIN), and healthy controls showing difference in vessel numbers. *P < 0.05, ***P < 0.001.

The mean total number of arteries and veins was significantly reduced by 24% and 29%, respectively, in PWA compared with controls (arteries = 15.5 compared to 20.3, *P <* 0.001 and veins = 12.9 compared to 18.2, *P <* 0.001, respectively; Table 1, Fig. 3). When categorized by branch, we observed that these changes were specifically due to significantly fewer secondary and tertiary arteries (secondary = 6.42 compared to 9.24, *P <* 0.001 and tertiary = 0.92 compared to 3.09, *P <* 0.001, respectively) and veins (secondary = 5.38 compared to 8.44, *P <* 0.001 and tertiary = 1.08 compared to 2.29, *P =* 0.026, respectively) in PWA as compared with controls, indicating reduced branching. Tertiary arteries and veins were only detected in 54% (13/24) and 58% (14/24) of PWA, respectively, which was less than in controls – 91% (31/34), Chi-square = *P* = 0.001) and 82% (28/34), *P* = 0.044, respectively. Numbers of PWIIN in whom tertiary arteries and veins could be detected was also lower – 60% (6/10), *P* = 0.018 and 80% (8/10) *P* = 0.865, respectively. The mean diameter values presented later are therefore only those participants in whom tertiary vessels were detected.

**Table 1.** Mean (± SD) and Statistical Comparisons of the Number of the Detected Vessels in Participants With Albinism (PWA) (*n* = 24), Participants With Idiopathic Infantile Nystagmus (PWIIN) (*n* = 10), and Controls (*n* = 34)

				Statistical Comparisons				
	Means and Standard Deviations			ANOVA		Post Hoc		
	Albinism	Idiopathic	Controls			Alb vs.	IIN vs.	Alb vs.
Arteries	Mean ± SD	Mean ± SD	Mean ± SD	F	*P* Value	Ctrl	Ctrl	IIN
*Primary*	8.21 ± 1.84	7.60 ± 0.84	7.68 ± 1.70	0.855	0.430	NS	NS	NS
*Secondary*	6.42 ± 2.41	8.60 ± 2.07	9.24 ± 2.70	8.999	**<0.001**	**<0.001**	NS	NS
*Tertiary* ^*^	0.92 ± 1.32	2.00 ± 1.70	3.09 ± 2.40	8.443	**<0.001**	**<0.001**	NS	NS
*Total*	15.54 ± 3.99	18.30 ± 2.91	20.29 ± 4.39	9.570	**<0.001**	**<0.001**	NS	NS
**Veins**								
*Primary*	6.42 ± 1.32	7.30 ± 1.77	7.29 ± 1.34	3.069	0.053	NS	NS	NS
*Secondary*	5.38 ± 2.02	6.40 ± 1.78	8.44 ± 2.19	15.920	**<0.001**	**<0.001**	**0.024**	NS
*Tertiary* ^†^	1.08 ± 1.25	1.30 ± 1.64	2.29 ± 1.93	4.031	**0.022**	**0.026**	NS	NS
*Total*	12.88 ± 3.13	15.10 ± 3.18	18.21 ± 3.16	20.491	**<0.001**	**<0.001**	**0.024**	NS

Significant differences (*P* < 0.05) shown in bold font.

* Albinism *n* = 13; idiopathic *n* = 8; and controls *n* = 31.

† Albinism *n* = 14; idiopathic *n* = 6; and controls *n* = 28.

The patterns of significant differences compared with controls were similar for the two FH subgroups. See Supplementary Figures S2 and S3 which show boxplots of these data. Average number of arteries was significantly less in both moderate and severe subgroups (14.83 and 16.25, respectively, compared to 20.29 in controls, *P <* 0.001) and veins (12.67 and 13.08, respectively, compared with 18.21 in controls, *P <* 0.001). The only discordance between the two subgroups compared with controls, was that participants with severe FH had a significant reduction (*P =* 0.044) of 15% less primary veins, whereas the moderate FH subgroup did not (6.17 and 6.67, respectively, compared with 7.29 in controls; Table 2).

**Table 2.** Mean (± SD) and Statistical Comparisons of the Number of the Detected Vessels of FH, 1 and 2 (*n* = 12), 3 and 4 (*n* = 12), and Controls (*n* = 34)

				Statistical Comparisons				
	Means and Standard Deviations			ANOVA		Post Hoc		
	Moderate FH	Severe FH	Controls			1 and 2	3 and 4	1 and 2
Arteries	Mean ± SD	Mean ± SD	Mean ± SD	F	*P* Value	vs. Ctrl	vs. Ctrl	vs. 3 and 4
*Primary*	7.75 ± 1.86	8.67 ± 1.78	7.68 ± 1.70	1.474	0.238	NS	NS	NS
*Secondary*	6.33 ± 1.97	6.50 ± 2.88	9.24 ± 2.70	8.236	**<0.001**	**<0.001**	**0.009**	NS
*Tertiary* ^*^	0.75 ± 0.87	1.08 ± 1.68	3.09 ± 2.40	8.016	**<0.001**	**<0.001**	**0.015**	NS
*Total*	14.83 ± 3.38	16.25 ± 4.56	20.29 ± 4.39	9.152	**<0.001**	**<0.001**	**0.019**	NS
**Veins**								
*Primary*	6.67 ± 1.50	6.17 ± 1.11	7.29 ± 1.34	3.482	**0.038**	NS	**0.044**	NS
*Secondary*	5.08 ± 2.19	5.67 ± 1.87	8.44 ± 2.19	14.776	**<0.001**	**<0.001**	**<0.001**	NS
*Tertiary* ^†^	0.92 ± 1.08	1.25 ± 1.42	2.29 ± 1.93	3.701	**0.031**	NS	NS	NS
*Total*	12.67 ± 2.99	13.08 ± 3.37	18.21 ± 3.16	19.929	**<0.001**	**<0.001**	**<0.001**	NS

FH, foveal hypoplasia (grading).

Significant differences (*P* < 0.05) shown in bold.

* Moderate FH *n* = 6, severe FH *n* = 7, and controls *n* = 31.

† Moderate FH *n* = 6, severe FH *n* = 8, and controls *n* = 28.

#### Participants With Idiopathic Infantile Nystagmus

In PWIIN, the total number of veins was 17% lower than controls (15.1 vs. 18.2, respectively; *P =* 0.024). This was mainly due to a significant reduction of 24% in the number of secondary veins compared with controls (6.40 vs. 8.44, respectively; *P =* 0.024; see Table 1), again indicating reduced branching.

Comparison of individual quadrants between the groups did not yield any significant differences.

### Vessel Diameter

#### Participants With Albinism

**Figure 4.**
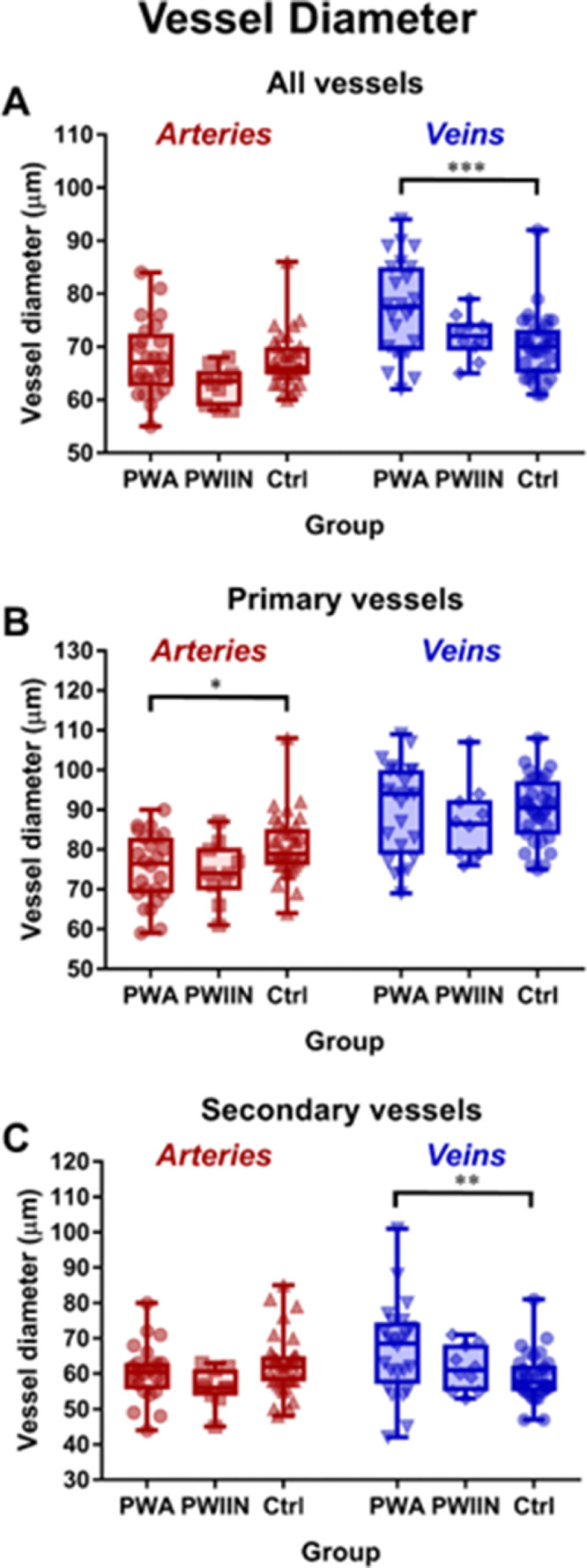
Box and whisker plots of vessel diameters of participants with albinism (PWA), Participants with idiopathic infantile nystagmus (PWIIN) and controls (Ctrl). *P < 0.05, **P < 0.01, ***P < 0.001.

Mean diameters of primary arteries of PWA (Table 3, Fig. 4) were significantly reduced (by 7%) compared with controls (75.39 µm compared to 80.88 µm, respectively; *P =* 0.043). Conversely, mean diameters of veins were significantly greater than in PWA compared with controls (11% larger in PWA; 77.56 µm, compared to 70.10 µm, respectively; *P <* 0.001). This was mainly due to changes in the secondary vein subgroup (13% larger in PWA; 66.72 µm, compared to 59.01 µm, respectively; *P =* 0.009).

**Table 3.** Mean (± SD) and Statistical Difference of the Diameter of the Detected Vessels in Participants With Albinism (PWA, *n* = 24), With Idiopathic Infantile Nystagmus (PWIIN, *n* = 10), and Controls (*n* = 34)

				Statistical Comparisons				
	Means (µ) and Standard Deviations			ANOVA		Post Hoc		
	Albinism	Idiopathic	Controls			Alb vs.		Alb vs.
Arteries	Mean ± SD	Mean ± SD	Mean ± SD	F	*P* Value	Ctrl	IIN vs. Ctrl	IIN
*Primary*	75.39 ± 8.80	74.45 ± 7.52	80.88 ± 7.92	4.256	**0.018**	**0.043**	NS	NS
*Secondary*	60.45 ± 7.91	56.30 ± 5.21	62.47 ± 8.45	2.419	0.097	NS	NS	NS
*All*	67.84 ± 6.89	62.76 ± 3.43	67.49 ± 5.04	3.271	**0.044**	NS	NS	NS
**Veins**								
*Primary*	90.27 ± 11.72	86.65 ± 9.45	90.03 ± 8.13	0.552	0.579	NS	NS	NS
*Secondary*	66.72 ± 13.11	61.53 ± 6.37	59.01 ± 6.50	4.786	**0.012**	**0.009**	NS	NS
*All*	77.56 ± 8.98	71.90 ± 4.15	70.10 ± 6.03	8.059	**<0.001**	**<0.001**	NS	NS

Significant differences (*P* < 0.05) shown in bold.

The severe FH albinism subgroup showed significantly thicker secondary veins than both moderate FH (71.55 µm compared to 61.89 µm, respectively; *P =* 0.042) and controls (71.55 µm vs. 59.01 µm, respectively; *P <* 0.001; see Supplementary Fig. S3). It is also notable that whereas the mean diameter of veins of PWA as a whole was significantly greater than that of controls (77.56 µm compared to 70.10 µm in controls, respectively; *P <* 0.001), the difference was more pronounced in the severe FH subgroup (79.36 µm, *P <* 0.001) whereas not significant with the moderate FH subgroup (75.75 µm; Table 4).

**Table 4.** Mean (± SD) and Statistical Difference of the Diameter of the Detected Vessels of FH, 1 and 2 (*n* = 12), 3 and 4 (*n* = 12), and Controls (*n* = 34)

				Statistical Comparisons				
	Means (µ) and Standard Deviations			ANOVA		Post Hoc		
	Moderate FH	Severe FH	Controls			1 and 2	3 and 4	1 and 2
Arteries	Mean ± SD	Mean ± SD	Mean ± SD	F	*P* Value	vs. Ctrl	vs. Ctrl	vs. 3 and 4
*Primary*	74.87 ± 8.86	75.90 ± 9.11	80.88 ± 7.92	3.077	0.054	NS	NS	NS
*Secondary*	58.94 ± 6.15	61.96 ± 9.38	62.47 ± 8.45	0.828	0.442	NS	NS	NS
*All*	66.89 ± 6.58	68.79 ± 7.34	67.49 ± 5.04	0.336	0.716	NS	NS	NS
**Veins**								
*Primary*	88.00 ± 10.56	92.55 ± 12.81	90.03 ± 8.13	0.658	0.522	NS	NS	NS
*Secondary*	61.89 ± 12.16	71.55 ± 12.66	59.01 ± 6.50	8.027	**<0.001**	NS	**<0.001**	**0.042**
*All*	75.75 ± 8.49	79.36 ± 9.45	70.10 ± 6.03	7.944	**<0.001**	NS	**<0.001**	NS

FH grading is carried out using OCT. Significant differences (*P* < 0.05) shown in bold.

#### Participants With Idiopathic Infantile Nystagmus

The PWIIN group did not demonstrate any significant differences in vessel diameter with either the control or PWA groups by vessel type or branching. However, when subdivided into quadrant, it was found the mean diameter of the arteries in the PWIIN group was significantly reduced in the superior temporal quadrant compared to controls (63.61 µm vs. 73.32 µm, *P =* 0.039) by 13%.

### Retinal Thickness and Spherical Equivalent Correlation to Vessel Parameters

Retinal thickness measurements at a horizontal distance of 6.3 mm from the optic disc center could be derived for 18 of the 24 PWA, with motion artefact due to nystagmus being the main cause of poor data that could not be analyzed. The results of Pearson correlation analysis (*r* and *P* values) are presented in Supplementary Table S2. There were no significant correlations between retinal thickness or any vessel parameter. Two parameters however, the number of secondary arteries and the total number of veins, demonstrated *P* values of *P* < 0.1 and *r* > 0.4.

The results of Pearson correlation analysis between vessel parameters and refractive error (*r* and *P* values) are presented in Supplementary Table S3. There were no significant correlations between spherical equivalent or any vessel parameter.

## Discussion

In this prospective, cross-sectional observational study, a quantitative analysis of vessel structure was performed for the first time in PWA and PWIIN in comparison with controls, comparing vessels counts and diameters for the various orders of vessel branching. In addition, the relationship between the degree of FH in PWA and vessel structure was also explored.

### Participants With Albinism 

PWA demonstrated a significant reduction in the total number of arteries and veins, as well as a reduction in the diameter of primary arteries, compared with controls. A relative diminishing of sequential branching of vessels in PWA compared with controls was noted; primary arteries and veins did not show any significant difference in vessel number, in contrast to secondary arteries and veins which were significantly reduced in PWA (Fig. 3^4^C), and tertiary arteries and veins being less than one third and one half that of controls, respectively.

Anatomically, the human retinal vascular system comprises of a superficial (inner), intermediate, and deep vascular plexus.^18^ The superficial plexus contains arteries and veins, whereas the deeper vascular beds comprise primarily of capillary-sized vessels.^19^ It is likely, therefore, that the visible vessels detected by the ARIA software are part of the superficial plexus.^27^ This superficial plexus is formed primarily by the process of vasculogenesis, which represents de novo establishment of the primordial vascular scaffold.^28^^,^^29^ Therefore, these findings indicate a disruption of vasculogenesis of the visible retinal vasculature in PWA.

In healthy eyes, the vascular network emerges from the optic nerve head at 16 weeks gestational age (WG) and spreads across the inner retina, reaching the temporal periphery at 40 WG.^18^ This temporal change may be relevant because the process of sprouting, remodeling, and branching of the vascular network from 25 WG is labeled angiogenesis, which thus may also be impaired in albinism.^30^ It is important to point out that in contrast to vasculogenesis, angiogenesis is generally associated with deeper plexus development, although a degree of both temporal and physiological overlap may exist between these two phases.^18^^,^^30^

One possible reason for these findings is reduced oxygen demand resulting from underdevelopment of the neural retinal network in albinism.^19^ In albinism, there is underdevelopment of the neural network as evidenced by FH and optic nerve abnormalities.^8^^,^^9^ In normal development, neuronal activity is the main precipitant of physiological hypoxia in the retina, which then induces vascular development through the production of factors such as vascular endothelial growth factor (VEGF) by glial cells.^19^ The superficial vascular plexus is anatomically aligned with retinal ganglion cells, which release platelet-derived growth factor (PDGF) to cause proliferation of astrocytes, which then produce a gradient of VEGF in order to stimulate blood vessel growth. Tip cells have been shown to use multiple neuronal guidance receptors to steer angiogenesis in order to match metabolic needs to that of vascular supply.^18^

In contrast to arteries, secondary venous branches were significantly thicker than in controls. We may speculate the reason for this upon their differing physiology. One key difference is that retinal arteries are lined with smooth muscle which enables autoregulation of blood flow.^19^ Retinal veins, due to their greater compliancy, may dilate as a result.^31^

The underdevelopment of the neural network in albinism is evident from FH, which resembles the immature fovea.^32^ In addition, the pattern of chiasmal misrouting in albinism points to delayed retinal development because retinal ganglion cell axons reaching the chiasm later in visual development are more likely to be contralateral.^33^ Abnormal retinal development in albinism in humans has been investigated in vivo in a developmental study by Lee et al. (2015), who reported delayed inner retinal layer migration, elongated photoreceptor layers, and reduced perifoveal retinal thickness.^34^ PWA are known to lack a foveal avascular zone (FAZ), whose size is likely causally correlated with foveal pit morphology.^35^^,^^36^ The link between melanin synthesis to the FAZ is, however, less clear. Despite studies showing a correlation between the size of the FAZ and macular pigment density,^36^ a direct causal link, such as that of antiangiogenic properties of pigment, has not been demonstrated. L-DOPA bound to the RPE GPR143 receptor stimulates PEDF production in OA1, whereas pigmentation is not necessarily dimished.^12^^,^^37^ PEDF functions as an anti-angiogenic factor, which in turn downregulates VEGF. It is believed that a mix of anti-angiogenic factors, including PEDF as well as axon guiding factors, play a role in creation and maintenance of the FAZ.^38^ Indeed anti-VEGF treatment in macular degeneration has been correlated with an increase in the size of the FAZ.^39^

### Participants With Idiopathic Infantile Nystagmus 

The IIN group displayed a similar pattern to albinism both in the number and the diameter of secondary and tertiary vessels, whose figures generally lay between that of the PWA and controls, although the differences were not significantly different in this smaller sample to either the PWA or the control group. An exception was the secondary venous quantity where it was significantly less in PWIIN than controls.

There are anatomic, clinical, and genetic links between IIN and albinism. Similar but milder structural and functional abnormal retinal development in IIN, as found in albinism, has been shown in association with *FRMD7* mutations.^40^ Using OCT, Thomas et al. (2014) have shown mild degrees of FH and greater central retinal thickness in nystagmus associated to *FRMD7* mutations.^5^ *FRMD7* also have abnormal optic nerve head morphology,^9^ and patients with IIN have been known to lack a FAZ.^41^ Clinically, the primarily conjugate horizontal nystagmus of albinism may resemble that of IIN, albeit with significantly worse visual acuities.^4^ Furthermore Volk et al*.* (2021) reported how mutations in l‑dopachrome tautomerase (DCT), a key enzyme in melanin synthesis, can produce variable phenotypes resembling either IIN or albinism.^42^ This suggests that retinal neuronal maldevelopment in IIN is the underlying pathology, causing disrupted vascular development albeit with less severe manifestations than in albinism.

As of yet, the mechanisms underlying nystagmus generation in INS remains elusive, although the presence of sensory deficits in FRMD7-related IIN along with other INS more obviously associated with afferent deficits, points to abnormal sensory inputs being a common underlying cause. It is possible that the oculomotor system responds to reduced sensory inputs during visual system development by ramping up the gain of feedback control circuits. Abnormal motion selectivity in retina in mice due to altered organization of neural networks caused by FRMD7 mutations also raises the possibility that nystagmus could be driven by abnormal inputs from the retina rather than as high gain oculomotor response to reduced inputs.^43^ In albinism, both these options are a possibility with FH leading to reduced central vision with mis-wired retinal ganglion cells clearly indicating changes in wiring of the peripheral retina.

A limitation of this study was that a relatively small area of the retina was analyzed. Therefore, our findings may not be representative of all retinal vasculature. Another weakness was that the study only analyzed data from 10 PWIIN and therefore may not be fully representative of this cohort as well as being insufficiently powered for statistical analysis. FH grading was carried out by only a single investigator, although inter-assessor reliability in grading FH has been shown to be very high.^44^ Additionally, the use of an automated image analysis method carries a potential for systematic bias if image characteristics differ between each cohort. In particular, the impact of hypopigmentation in PWA and the motion artifact caused by nystagmus on vessel wall detection, and thus vessel diameter measurements, is uncertain. The issue of choroidal vasculature being inadvertently detected may be addressed by a segmentation algorithm utilizing OCT angiography to automatically exclude choroidal vessels.

No correlations existed between refractive error and vessel parameters in our data nor did adjusting for refractive error change statistical outcomes. However, spherical equivalent can be taken as an approximate surrogate measure for axial length, and a more robust assessment could be achieved by calibrating fundus images based on axial length, for which data were not available for this study.

## Summary

On color fundus imaging, PWA and PWIIN demonstrated reduced retinal vessel quantity and arterial diameter as compared with controls, which indicates underdevelopment of the superficial retinal vasculature. Venous diameter was, however, increased. Development of the retinal vascular network is guided by the neuronal network, which is also known to be disrupted in albinism and IIN. Therefore, this opens another avenue of investigation in determining the molecular pathophysiology of INS.

## Supplementary Material

Supplement 1
