# Supplementary material for: Abnormal Retinal Vessel Architecture in Albinism and Idiopathic Infantile Nystagmus
Source: Invest Ophthalmol Vis Sci. 2022 May 26;63(5):33. doi: 10.1167/iovs.63.5.33 (PMC9150830; doi:10.1167/iovs.63.5.33)
Supplement: Supplement 1 [file iovs-63-5-33_s001.pdf]

Supplementary Table 1. *Baseline characteristics of PWA, PWIIN and controls.*

**Group**	**Albinism** (n=24)	**IIN** (n=10)	**Controls** (n=34)
Male : female ratio	1.7 : 1	2.3 : 1	1.8 : 1
	Mean ± SD	Mean ± SD	Mean ± SD
Age (years)	33.8 ± 14.2	31.8 ± 17.0	33.1 ± 14.5
Spherical equivalent (Dioptres)	0.51 ± 2.61	-0.73 ± 2.09	-0.72 ± 1.27
Visual acuity (logMAR)	0.52 ± 0.33	0.11 ± 0.09	0.04 ± 0.06
Foveal hypoplasia grade*	2.67 ± 1.27	0.60 ± 0.52	-

PWA – Participant with Albinism. PWIIN – Participants with idiopathic infantile nystagmus.

**Based on the structural grading system developed by Thomas et al (2011)*[21]

ANOVA analysis with Bonferroni correction showed no statistical differences between the groups for any domains except for mean visual acuity (p=0.001) for which post-hoc analysis showed the albinism group to be significantly poorer than IIN (p=0.001) and controls (p=0.001).

Supplementary Table 2. *Pearson correlation analysis of retinal thickness* to mean vessel count and mean vessel thickness of primary, secondary, tertiary and total arterioles and venules, in a cohort of PWA (n=18)*

	Number of vessels		Vessel diameter	
	r	***P***	***r***	***P***
**Arterioles**				
Primary	0.337	0.172	-0.047	0.852
Secondary	0.403	0.097	-0.303	0.222
Tertiary**	0.109	0.667	-	-
Total	0.162	0.520	-0.304	0.219
**Venules**				
Primary	-0.279	0.262	0.149	0.555
Secondary	-0.035	0.892	-0.046	0.856
Tertiary**	-0.158	0.531	-	-
Total	0.411	0.090	0.079	0.755

PWA – Participant with Albinism.

** Retinal thickness was measured in the albinism group from OCT B-scan images from the temporal aspect of the optic nerve head, at a horizontal distance of 6.3mm from the optic disc centre*

*** Insufficient data to complete analysis*

Supplementary Table 3. *Pearson correlation analysis of spherical equivalent to mean vessel count and mean vessel thickness of primary, secondary, tertiary and total arterioles and venules, in a cohort of PWA (n=18)*

	Number of vessels		Vessel diameter	
	r	***P***	***r***	***P***
**Arterioles**				
Primary	0.142	0.508	0.038	0.860
Secondary	0.104	0.630	0.143	0.504
Tertiary*	0.086	0.688	-	-
Total	0.157	0.464	0.076	0.723
**Venules**				
Primary	-0.121	0.573	0.126	0.557
Secondary	0.118	0.582	0.126	0.556
Tertiary*	0.100	0.641	-	-
Total	0.066	0.761	0.255	0.229

PWA – Participant with Albinism.

** Insufficient data to complete analysis*

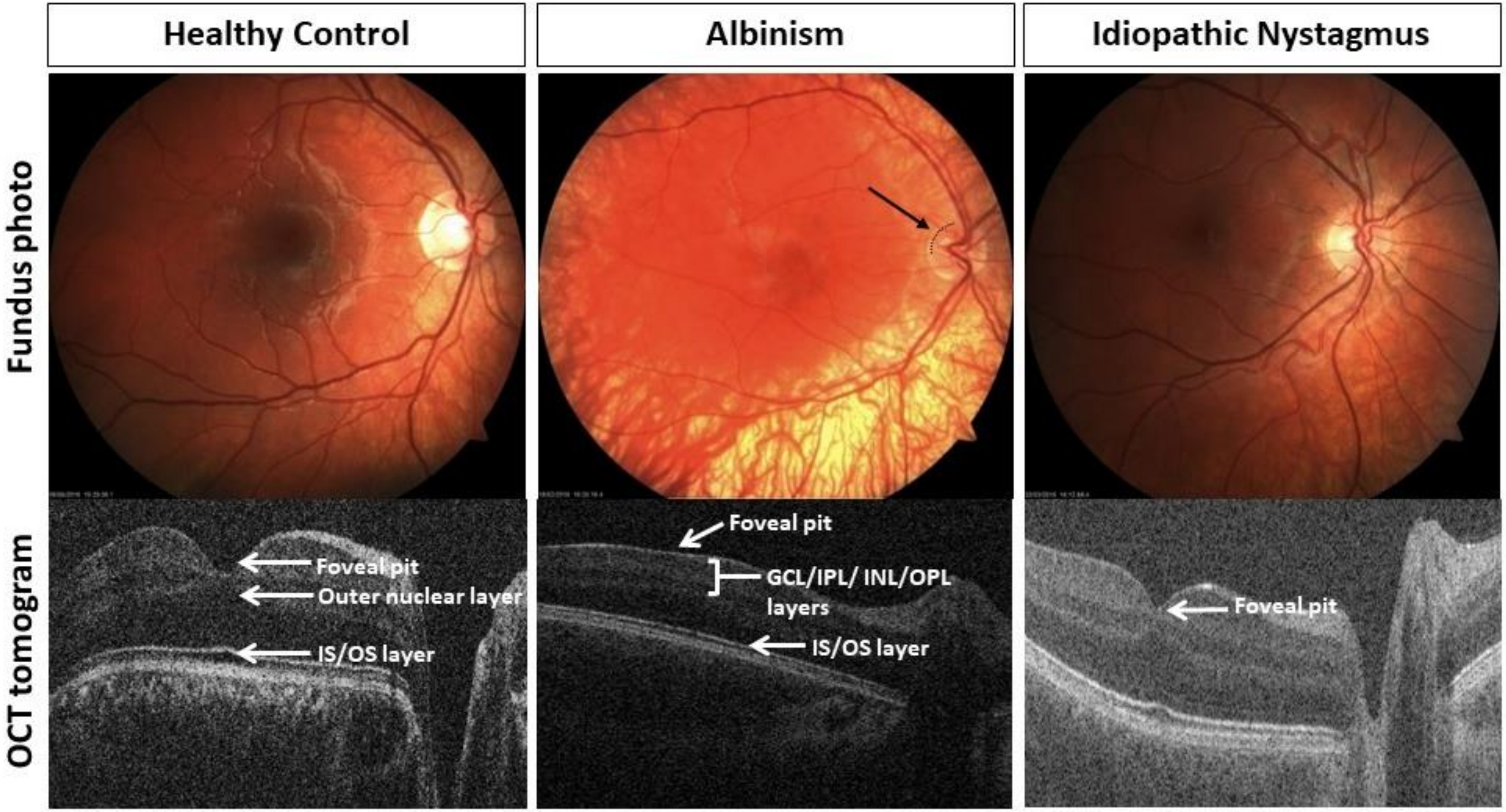

Supplementary Figure 1: *Fundus photography and horizontal optical coherence B-scans of the fovea and optic discs of age-, gender- and ethnicity matched participants.*

Fundus photography (top row) shows normal foveal reflex on heathy control and participants with idiopathic infantile nystagmus (PWIIN). The albinism participant demonstrates small, cupless disc, initial nasal deflection of retinal vessels (situs inversus), double ring sign (see arrow and dotted line), poorly discernible macula and prominent choroidal vasculature. Optical coherence tomography (OCT) images (bottom row) shows deep foveal pit and updrift of the ONL and IS/OS in the fovea of the control; the albinism participant has a flat pit, continuation of the GCL, INL, IPL and OPL in the fovea with a flat IS/OS line (grade 4 foveal hypoplasia); the PWIIN had a small pit, continuation of the GCL and INL, and a preserved updrift of the IS/OS line (grade 1 foveal hypoplasia)

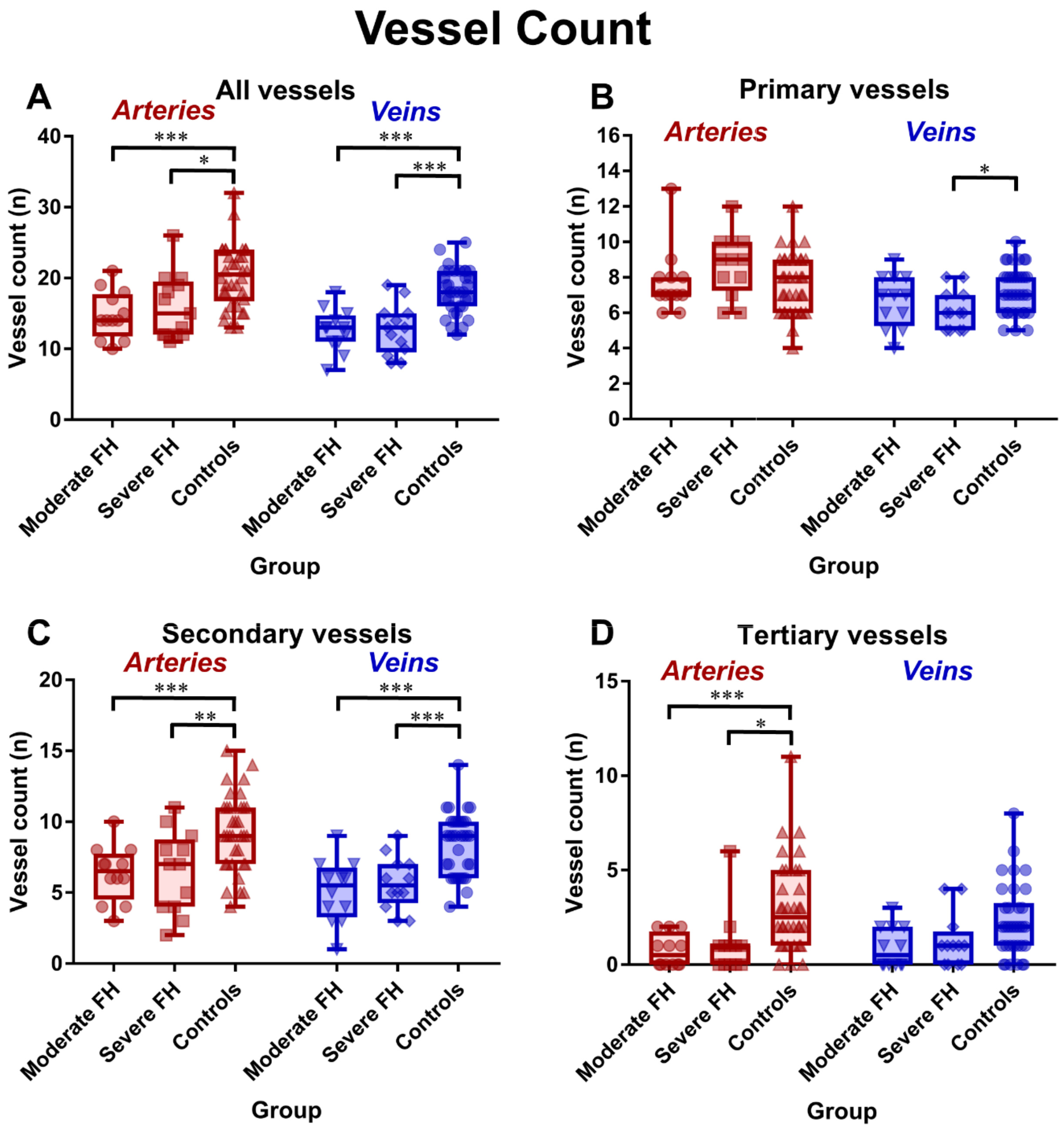

Supplementary Figure 2*: Box and whisker plots of vessel counts of participants with albinism, with moderate foveal hypoplasia, severe foveal hypoplasia and Control subgroups*

** p<0.05; ** p<0.01, *** p<0.001*

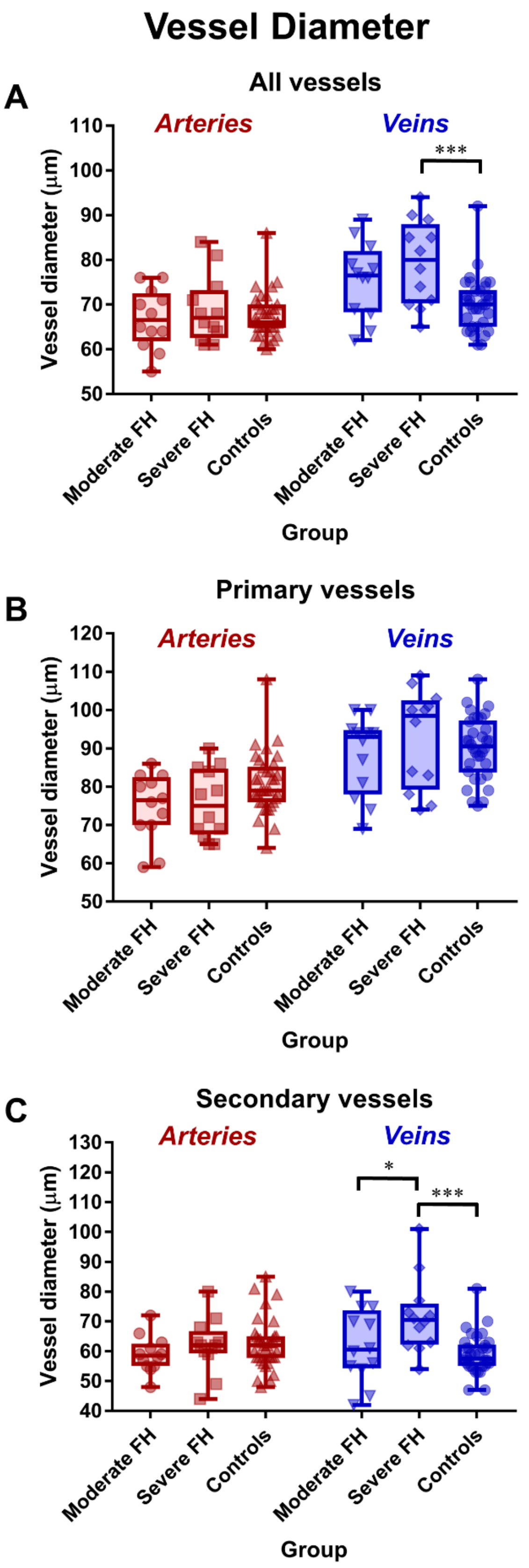

Supplementary Figure 3*: Box and whisker plots of vessel diameters of participants with albinism, with moderate foveal hypoplasia, severe foveal hypoplasia and Control subgroups*

** p<0.05; *** p<0.001*
